# Magnitude of Alloresponses to MHC Class I/II Expressing Human Cardiac Myocytes is Limited by their Intrinsic Ability to Process and Present Antigenic Peptides

**DOI:** 10.1080/10446670310001642410

**Published:** 2003

**Authors:** J. Bruce Sundstrom, Kimberley C. Jollow, Veronique Braud, Francois Villinger, Andrew J. McMichael, E. Clinton Lawrence, Edwin W. Ades, Aftab A. Ansari

**Affiliations:** 1 Department of Pathology and Laboratory Medicine Emory University School of Medicine Atlanta GA USA; 2 Molecular Immunology Group Institute of Molecular Medicine John Radcliffe Hospital Oxford OX3 9DU UK; 3 Department of Surgery Emory University School of Medicine Atlanta GA USA; 4 Centers for Disease Control and Prevention Atlanta GA USA

## Abstract

In this investigation we have explored the relationship between the weak allogenicity of cardiac myocytes and their capacity to present allo-antigens by examining the ability of a human cardiac myocyte cell line (W-1) to process and present nominal antigens. W-1 cells (HLA-A*0201 and HLA-DR β1*0301) pulsed with the influenza A matrix 1 (58-66) peptide (M1) were able to serve as targets for the HLA-A*0201 restricted CTL line PG, specific for M1-peptide. However, PG-CTLs were unable to lyse W-1 target cells infected with a recombinant vaccinia virus expressing the M1 protein (M1-VAC). Pretreatment of these M1-VAC targets with IFN-γ partially restored their ability to process and present the M1 peptide. However, parallel studies demonstrated that IFN-γ pretreated W-1's could not process tetanus toxin (TT) or present the TT(830-843) peptide to HLA-DR3 restricted TT-primed T cells. Semi-quantitative RT-PCR measurements revealed significantly lower constitutive levels of expression for MHC class I, TAP-1/2, and LMP-2/7 genes in W-1s that could be elevated by pretreatment with IFN-γ to values equal to or greater than those expressed in EBV-PBLs. However, mRNA levels for the genes encoding MHC class II, Ii, CIITA, and DMA/B were markedly lower in both untreated and IFN-γ pretreated W-1s relative to EBV-PBLs. Furthermore, pulse-chase analysis of the corresponding genes revealed significantly lower protein levels and longer half-life expression in W-1s relative to EBV-PBLs. These results suggest that weak allogenicity of cardiac myocytes may be governed by their limited expression of MHC genes and gene products critical for antigen processing and presentation.


*Clinical & Developmental Immunology*, June–December 2003 Vol. 10 (2–4), pp. 213–226

Taylor & Francis healthsciences

### Magnitude of Alloresponses to MHC Class I/II Expressing Human Cardiac Myocytes is Limited by their Intrinsic Ability to Process and Present Antigenic Peptides*

J. BRUCE SUNDSTROM [a], KIMBERLEY C. JOLLOW[a], VERONIQUE BRAUD[b], FRANCOIS VILLINGER[a], ANDREW J. McMICHAEL[b], E. CLINTON LAWRENCE[c], EDWIN W. ADES[d] and AFTAB A. ANSARI[a,†]

[a]*Department of Pathology and Laboratory Medicine, Emory University School of Medicine, Atlanta, GA, USA;* [b]*Molecular Immunology Group, Institute of Molecular Medicine, John Radcliffe Hospital, Oxford OX3 9DU, UK;* [c]*Department of Surgery, Emory University School of Medicine, Atlanta, GA, USA;* [d]*Centers for Disease Control and Prevention, Atlanta, GA, USA*

In this investigation we have explored the relationship between the weak allogenicity of cardiac myocytes and their capacity to present allo-antigens by examining the ability of a human cardiac myocyte cell line (W-1) to process and present nominal antigens. W-1 cells (HLA-A*0201 and HLA-DR β1*0301) pulsed with the influenza A matrix 1 (58-66) peptide (M1) were able to serve as targets for the HLA-A*0201 restricted CTL line PG, specific for M1-peptide. However, PG-CTLs were unable to lyse W-1 target cells infected with a recombinant vaccinia virus expressing the M1 protein (M1-VAC). Pretreatment of these M1-VAC targets with IFN-γ partially restored their ability to process and present the M1 peptide. However, parallel studies demonstrated that IFN-γ pretreated W-1's could not process tetanus toxin (TT) or present the TT(830-843) peptide to HLA-DR3 restricted TT-primed T cells. Semi-quantitative RT-PCR measurements revealed significantly lower constitutive levels of expression for MHC class I, TAP-1/2, and LMP-2/7 genes in W-1s that could be elevated by pretreatment with IFN-γ to values equal to or greater than those expressed in EBV-PBLs. However, mRNA levels for the genes encoding MHC class II, Ii, CIITA, and DMA/B were markedly lower in both untreated and IFN-γ pretreated W-1s relative to EBV-PBLs. Furthermore, pulse-chase analysis of the corresponding genes revealed significantly lower protein levels and longer half-life expression in W-1s relative to EBV-PBLs. These results suggest that weak allogenicity of cardiac myocytes may be governed by their limited expression of MHC genes and gene products critical for antigen processing and presentation.

*Keywords*: Cardiac transplantation; Allorecognition; MHC genes; Antigen processing

### INTRODUCTION

The molecular basis for chronic immune-mediated rejection of solid human organ allografts continues to be defined (Hayry *et al.*, 1995). In the case of cardiac allografts, such chronic rejection is associated with loss of myocytes which is reasoned to be secondary to lysis mediated by donor specific "alloactivated" host derived graft infiltrating lymphocytes (GIL's) (Duquesnoy and Demetris, 1995). It is now established that in order for T cell activation to occur by either the direct or indirect pathway at least two signals are required (Bretscher, 1992). The first signal is engendered by cognate interactions between the TCR on responder T cells and the appropriate peptide bearing MHC molecules displayed on the surface of the allostimulator cells. The second signals develop and act synergistically with the first signal and are mediated by the engagement of cell surface expressed cellular adhesion and co-stimulatory molecules (CAM's/CSM's) with their respective ligands. Some of these CAM's/CSM's and their ligands are constitutively expressed and others are induced by agents such as cytokines. The quality and density of these receptors and their cognate ligands that comprise CAM's/CSM's expressed by allogeneic cells have been shown to influence the type and relative strength of the second signals generated (De Franco and Coffman, 1995). In addition to the requirement for these two signals, data has been presented to support the concept that the strength (magnitude) of the allo-immune response is in part a reflection of the number of antigen specific responder T cell clones that become activated following recognition

*This research was supported by NIH-1RO1-HL47272 and HL44203.

†Corresponding author. Address: Department of Pathology and Laboratory Medicine, Room 2309 WMB, 1639 Pierce Dr., Emory University School of Medicine, Atlanta, GA, 30322, USA. Tel.: +1-404-712-2834. Fax: +1-404-712-1771. E-mail: pathaaa@emory.edu

ISSN 1740-2522 print/ISSN 1740-2530 online © 2003 Taylor & Francis Ltd
DOI: 10.1080/10446670310001642410

of their respective peptide bearing MHC allo-antigens on the stimulator cells (Warrens, 1994). This concept implies that a certain threshold frequency of T cell clones must be activated for an allogeneic response to be measured by conventional techniques. Furthermore, detectable host T cell alloproliferative responses become dependent on the diversity of peptides that are presented by MHC molecules on the stimulator cells within the allograft. Therefore, the inherent limitations of a given cell lineage to induce allogeneic immune responses (allogenicity) may be dictated by the intrinsic ability of that cell to process and present diverse sets of antigenic peptides.

However, the intrinsic abilities of cells comprising the allograft to process and present antigenic peptides may vary with their physiological, tissue lineage, or developmental status. These characteristic potentials are most likely manifested in the differential regulation and expression of genes involved in the processing and presentation of antigenic peptides as well as the synthesis, transport and assembly of MHC Class I/II molecules to the cell surface. Besides the MHC molecules, these include but are not limited to the genes that encode CIITA, Ii, TAP-1/2, LMP-2/7 and DMA/B (Neefjes, 1995; Mach *et al.*, 1996). We have previously proposed that cells derived from distinct tissue lineages may be categorized as non-professional, semi-professional and professional antigen presenting cells (APC) based on their relative abilities to process and present antigens (Sundstrom and Ansari, 1995). This view is supported by our data derived from a series of studies utilizing primary cultures of various lineages of human cells including fetal human cardiac myocytes (FHCM's) and the surrogate FHCM cell line termed W-1 (Wang *et al.*, 1991). Our laboratory has shown that neither FHCM's nor the surrogate FHCM cell line, W-1, when pretreated with IFN-γ are able to induce primary or to trigger secondary alloresponses *in vitro*. This deficient allogenicity is not secondary to the lack of expression of stable peptide-associated MHC Class 1/II, the residual effects of IFN-γ, or the elaboration of suppressive cytokines, e.g. TGF-β (Ansari *et al.*, 1995a). Furthermore, W-1s do express functional costimulatory molecules, e.g. LFA-3, and ICAM-1, yet they are still unable to induce alloproliferative responses in resting T cells even after their reconstitution with a human glycosyl-phosphatidylinositol (GPI)-anchored B7-1 molecule (GPI-B7-1, manuscript in preparation). Therefore, in this investigation we chose to examine and compare the abilities of W-1s and EBV-PBLs to process and present nominal antigens to primed class II and class I restricted T cells. Our data revealed that in contrast to EBV-PBLs, IFN-γ pretreated MHC Class II expressing W-1s incubated with TT antigen or pulsed with the antigenic P2 peptide were unable to induce proliferative responses in TT primed T cells. When similar experiments were performed for the MHC Class I processing and presentation pathways, W-1s were able to present exogenoulsy added influenza A M1 peptide to the peptide-specific CTL cell line, PG. However, W-1s were able to process and present the native influenza A matrix 1 protein only after pretreatment with IFN-γ. These results prompted us to examine the regulation of genes involved in the MHC Class I/II antigen processing and presentation pathways in the W-1 cell line. We compared the levels of expression of MHC genes and their gene products in the W-1s, before and after pretreatment with IFN-γ, with constitutive levels in other professional and non professional APCs to see how they might correlate with their abilities to process and present antigenic peptides. Our data suggest that the functional limitations of W-1s to process antigenic peptides for presentation by MHC Class I or by Class II are related to both their relatively weak gene expression within the MHC locus and the prolonged turnover time of the corresponding gene products. The results of these investigations constitute the basis of this report.

### MATERIALS AND METHODS

### Preparation and Culture Conditions of Lymphocytes and Cell Lines

The surrogate FHCM cell line, W-1, was derived by SV40 large-T-antigen induced transformation of an enriched population of FHCM as previously described (Wang *et al.*, 1991). This cell line remains phenotypically stable with respect to MHC expression, cardiac myosin content, expression of insulin-like growth factor receptor type 1, thrombin receptors, $P_2Y$ purinoceptors, β-adrenergic receptors, and reactivity with immunochemical reagents that distinguish this cell line as being of cardiac myocyte origin and not of endothelial, epithelial, or smooth muscle origin. This cell line has been serologically typed for MHC Class I as HLA A2.A30, B17.B18; C3.C5,Bw4.Bw6 and molecularly typed for MHC Class II as DRβ1*0301, DRβ 1*1302, DRβ 3*0202, DRβ 3*0301, and DQβ1*0201 (Table I). The adherent W-1 cell line was cultured in HM-1 medium. HM-1 medium consists of equal volumes (38%) of BME and HAM's F10 nutrient mixture, 5% human AB serum, 20% FBS, 2 mM L-glutamine, bovine insulin (50 μg/ml, Sigma) and gentamicin (50 μg/ml). All sera were heat-inactivated at 56°C for 1 h prior to use. Primary and immortalized human lung (pulmonary) microvascular endothelial cells (Lawrence *et al.*, 1995; Waite-Rees *et al.*, 1996) and primary and immortalized human dermal microvascular endothelial cells (Ades *et al.*, 1992) that were used in control experiments were grown and maintained as previously described. Donor peripheral blood mononuclear cell (PBMC) responders were isolated by gradient centrifugation on LSM (Organon Teknika, Durham, NC) as previously described (Ansari *et al.*, 1995b). Preparation of enriched $CD3^+$ populations of responders was accomplished by subjecting PBMCs to negative selection over Human T-cell enrichment columns (R&D systems). The enriched T cell populations were shown to be greater

TABLE I Allelic Classification of HLA specificities for APCs and responder cells

| | | | | | | | |
|---|---|---|---|---|---|---|---|
| *A. Molecular classification of MHC class II specificities* | | | | | | | |
| APC/ responder | DRB1a | DRB1b | DRB3a | DRB3b | DRB5b | DQB1a | DQB1b |
| W-1 | 0301 | 1302 | 0202 | 0301 | | 0201 | |
| LW PBMC | 0301 | 1301/4 | 0101 | | | 0201 | 0603 |
| JS-EBV-PBL | 0301 | 1501/3/4 | 0202 | | 0101 | 0201/2 | 0602/3 |
| *B. Serological classification of MHC class I specificities* | | | | | | | |
| APC | Aa | Ab | APC | Bb | | | |
| W-1 | 2 | 30 | 17 | 18 | | | |
| LW-EBV-PBL | 2 | 24 | 8 | 62 | | | |
| JS-EBV-PBL | 2 | 30 | 18 | 51 | | | |

that 95% $CD3^+$ by flow microfluorometric analysis (FMF) using FITC-conjugated antibodies directed against human CD3 (data not shown). As a source of autologous stimulator cells, an Epstein Barr Virus transformed cell line was derived from this same donor's (LW) peripheral blood lymphocytes by incubating isolated PBMCs *in vitro* with supernatant fluid from the B95-8 marmoset cell line (Ansari *et al.*, 1995b). Another EBV-PBL cell line developed from donor JS, who also shares HLA-DR identity with W-1 (HLA-β1*0301, HLA-A2*0201), was used in some experiments for the purpose of control. For CTL experiments, the human PG CTL line was generated as previously described (Gotch *et al.*, 1987). This CTL line recognized the HLA-A2*0201 restricted influenza A matrix 1 epitope GILGFVFTL (corresponding to residues 58-66). The human lymphoblastoid cell line PG that was used as a source of autologous APCs in CTL experiments was grown in RPMI 1640 with 10% FCS.

### Preparation of Synthetic Peptides

The peptides listed in Table II were prepared by solid-phase synthesis on an Applied Biosystems (Foster City, CA) model 430A peptide synthesizer with phenyl-acetamidomethyl copoly (styrene/divinyl benzene) resins and *tert*-butyloxycarbonyl (*t*-Boc) amino acid protection strategies. Some peptide preparations were N-terminally biotinylated via a two β-alanine spacer using the linking agent hydroxybenzotriazol (HOBT). The crude peptides were purified by microbore reversed-phase high-pressure liquid chromatography (HPLC) on an Aquapore OD-300$C_{18}$ silica columns (1 by 250 mm) with a linear gradient of acetonitrile in 0.1% aqueous trifluoroacetic acid and stored at $-20$°C in lyopholized form as previously described (Shafer *et al.*, 1993). Due to the hydrophobic characteristics of the P2 peptide, solubilization with dimethyl sulfoxide ($<1$% v/v) was necessary for use *in vitro*. Therefore, for experiments using the soluble P2 peptide, control cultures were suspended in media containing the same concentration of DMSO. The influenza A matrix 1 (58-66) peptide, GILGFVFTL, was synthesized manually using fmoc chemistry. Cleavage and deprotection was done with TFA. The peptide was purified by ether precipitation followed by reversed-phase HPLC and was checked for purity ($>95$%) by HPLC. This peptide was also solubilized in DMSO for experiments as described above.

### Proliferation Assays

All *in vitro* cell proliferation assays were performed in triplicate wells in a volume of 0.2 ml of complete media in round-bottomed 96-well tissue culture plates. Complete culture media consisted of RPMI 1640 with 10% FBS, 2 mM L-glutamine, and gentamicin (50 μg/ml). Cocultures were incubated at 37°C and 7% $CO_2$ for five days, then each well was pulsed with 1 μg Ci/well of methyl- $^3$H thymidine (2 Ci/mmol, NEN, Boston, MA) for 16 h before harvesting and counting for 1 min on a LKB liquid scintillation counter.

### *Preparation of APC's and Antigen-primed T Cells*

For experiments measuring the abilities of cells to process and present TT antigen, LW-EBV-PBL or the W-1 cells (HLA-DRβ1*0301) were incubated overnight in complete media containing TT (1 Lf/ml). They were then washed twice, suspended at $10^6$ cells/ml (W-1's) or $2 \times 10^6$ cells/ml (LW-EBV-PBL) in complete media, then γ-irradiated (80 Gy). A total of $5 \times 10^4$ (W-1's) or $10^5$

TABLE II Synthetic peptides

| Synthetic peptide | Sequence | HLA-restriction(s) | References |
|---|---|---|---|
| TT 830-843 (P2) | QYIKANSKFIGITE | DR3, DR1, DRw15(2), DRw18(3), DR4Dw4, DRw11(5), DRw13(w6), DR7, DRw8, DR9, DRw52a, and DRw52b | (Panina-Bordignon *et al.*, 1 989; O'Sullivan *et al.*, 1991; Reece *et al.*, 1993) |
| Influenza A matrix 1 peptide 58-66 (M1) | GILGFVFTL | A2 | (Bednarek *et al.*, 1991) |
| Influenza HA (306-318) | PKYVKQNTLKLAT | DR1, DR2, DR5, DR7 | (O'Sullivan *et al.*, 1991; Weber *et al.*, 1996) |

(autologous EBV-PBLs) of the antigen pulsed APCs were then mixed 1:1 (v/v) with $10^5$ unfractionated LW-PBMCs or T cell enriched LW responders and cocultured as described above. For experiments measuring the abilities of cells to present the P2 antigenic peptide, the appropriate APCs were suspended at $10^7$/ml in serum free PBS containing 100 μg/ml synthetic P2 and then incubated for 2 h at 37°C, then washed twice before placing in coculture with TT primed T cells. TT primed cells were prepared from an individual donor (LW) who shares HLADRβ1*0301 specificity with the W-1 cell line (Table I) and who had also received a recent (2 weeks) tetanus booster vaccination. Controls included with each experiment consisted of measurements of the comparative proliferative responses of unfractionated LW-PBMCs and highly enriched T cells that were collected before and after the TT immunization. In addition, antigen specificity of the response was determined with the use of KLH pulsed APCs. The results verified that the proliferative responses of TT-primed responders were secondary and specific for TT antigen (data not shown).

### Cytotoxicity Assays

The CTL activity of a $CD8^+$, HLA-A*0201 restricted human CTL cell line (PG) specific for the influenza A matrix 1 peptide, M1, was measured in a standard $^{51}Cr$ release assay as previously described (Morrison *et al.*, 1992). Target cells consisting of autologous PG lymphoblastoid cells or W-1s with and without pretreatment with IFN-γ were incubated at 37°C with peptide M58-66 at 5 μM or mock treated for 1 h, then washed and labeled with 100 μCi of $^{51}Cr$ for 1 h at 37°C. For other experiments, target cells were infected with recombinant vaccinia viruses M1-VAC or NP-VAC at 10 plaque-forming-units (PFU) per cell for 90 min. The target cells were then washed and incubated for 18 h in culture medium before labeling with $^{51}Cr$ as described. M1-VAC and NP-VAC encode the full length influenza A matrix 1 protein and the full length influenza A nucleoprotein, respectively. These constructs were generated as previously described (Gotch *et al.*, 1987; Shafer *et al.*, 1993).

For the CTL assay, a total of $5 \times 10^3$ targets were incubated with various effector-to-target ratios (E:T), medium alone (spontaneous release), or 5% Triton X-100 (maximum release). After 5 h the plates were spun down and the radioactivity (average cpm) from each of duplicate wells was determined. The percentage of specific lysis was calculated as [(release by CTL targets) − (spontaneous release)] × 100/[(maximum release) − (spontaneous release)]. The spontaneous release was consistently below 15%. The expression of vaccinia encoded genes in targets was checked by monitoring β-galactosidase expression following NP-VAC infection. A total of $2 \times 10^5$ infected cells were lysed with $H_2O$ then X-Gal was added to a final concentration of 6 mg/ml. The kinetic degradation of X-Gal by infected W-1 and PG target cells were identical (data not shown).

### Quantitation of the Expression of Genes Involved in the MHC I/II Antigen Processing and Presentation Pathways by Semi-quantitative RT-PCR

Quantitation of the relative constitutive and cytokine induced levels of mRNA expressed by the human MHC class I/II genes CIITA, Ii, DMA, DMB, LMP-2, LMP-7, TAP-1 and TAP-2 from the various tissue lineage cell lines was determined using a semi-quantitative RT-PCR method established by our laboratory (Villinger *et al.*, 1995).

#### *RNA Isolation*

Total cellular RNA was isolated from $2 \times 10^6$ cells from each tissue lineage using a guanidium thiocyanate-phenol chloroform single step method. A total of 20 μg of carrier salmon sperm DNA was then added to each of the RNA samples to facilitate precipitation with isopropanol.

#### *Reverse Transcription of Cellular mRNA*

Reverse transcription (RT) of RNA isolated from each sample dilution was performed in the following manner. Air-dried RNA pellets were resuspended in 20 μl of reverse transcription mixture (pH 8.5) consisting of 50 mM Tris, 6 mM $MgCl_2$, 40 mM KCl, 10 mM DTT, 0.01% Nonidet P-40, 50 μM random hexamers, 25 μM deoxynucleotide triphophates (dNTP), 3 U RNasin, and 30 U murine leukemia virus reverse transcriptase (Promega, Madison, WI). Reverse transcription of cellular mRNA into first strand cDNA was allowed to proceed at room temperature for 10 min and then at 42°C for 1 h followed by heat inactivation at 95°C for 5 min. The efficiency and sensitivity of each RT reaction was determined by amplification of GAPDH transcripts (25 cycles).

#### *PCR Amplification*

Oligonucleotide primer pairs (Table III) specific for each of the human MHC Class I/II genes were prepared according to published gene sequences. Amplification of aliquots of the cDNA reverse transcribed from each dilution of the sample RNA was performed in 50 μl of a reaction mixture containing 2.5 mM $MgCl_2$, 0.125 mM of each dNTP (dATP, dCTP, dGTP, and dTTP), 100 ng of each primer and 1 U of Taq polymerase in 1X Taq buffer (Perkin Elmer, Norwalk, CT). Samples were overlaid with mineral oil, (Sigma, St. Louis, MO) then subjected to 30 cycles of denaturation (95°C), annealing (60°C), and elongation (72°C) in a Perkin-Elmer thermocycler. Semi-quantitative evaluation of the amplified product was performed by parallel amplifications of ten-fold serial dilutions ($10^{-6}$ to $10^{-1}$ copies) of each respective cloned gene. Amplification products were separated by agarose-gel electrophoresis, blotted onto nylon membranes, then hybridized with digoxigenin (DIG)-labeled (Boehringer Mannheim Biochemical) internal oligonucleotide (Table III).

TABLE III List of oligonucleotide primers used for amplification in LDA-RT-PCR for MHC ClassI/ II gene mRNA transcripts

| Oligo | Primer site | Sequence (5′ to 3′) | Size of amplified fragment (bp) |
|---|---|---|---|
| CIITA | 5′* | GCTTGGCTCGTGTGCTTCCG | 515 |
| | 3′† | TCAGCAGAGCAAGATGTGGTTCA | |
| | IP‡ | TCCACATCGCCAGAGTCTCC | |
| Ii | 5′ | TGGTGACTCTGCTCCTCGCTG | 515 |
| | 3′ | CAGATCCTGCTTGGTCACACC | |
| | IP | CTTTCGGTGGAGCGTCAGTGG | |
| DMA | 5′ | CTAAAAGCTGGTTGGTAGCTCC | 859 |
| | 3′ | GCTGGCATCAAACTCTGGTCTG | |
| | IP | AGCATCTCCCTGTTCCTGAGCC | |
| DMB | 5′ | GGCATCTTTACAGAGCAGAGCATGA | 647 |
| | 3′ | GGTGTCCAGTCCCGAAGGATG | |
| | IP | GGCTAAATGGGAGAGGGTCTGGT | |
| LMP-2 | 5′ | GCACCAACCGGGGACTTACC | 413 |
| | 3′ | TGAAGCGCCTGCACTCCTCG | |
| | IP | GTTCAGCTGCTGATGCCCAA | |
| LMP-7 | 5′ | TCTAGTCTTCTGGTTGAAGCTGCG | 509 |
| | 3′ | CGAGTCCCATGTTCATCCACGT | |
| | IP | GCTGCCGACACTGAAATACGTT | |
| TAP-1 | 5′ | CCTCTCGCTGTTCCTGGTCC | 703 |
| | 3′ | GATTCCCACTTTCAGCAGCATACC | |
| | IP | GCAGGACAGCCCCAAACACC | |
| TAP-2 | 5′ | AGCAGGACCAGGTGAACAACAA | 654 |
| | 3′ | CAAACCTGCGAACGGTCTGC | |
| | IP | CGCAAGAGCACATTGGCATTTA | |

* Sense primer.
† Anti-sense probe.
‡ Internal probe (digoxigenin-labeled).

Detection and quantitation of PCR amplification products was accomplished by enzyme-linked immunoassay using a nonradioactive DIG DNA labeling and detection kit and by chemiluminescence (Boehringer Mannheim Biochemical). Semi-quantitative estimates of sample mRNA were performed by comparing the signals obtained from the test samples with those from serial dilutions of the cloned fragments. The signals were found to follow a linear range between $10^0$ and $10^9$ copies.

### Pulse Chase Experiments

Cells were metabolically labeled as follows. Confluent cultures were washed once in DPBS then incubated for 2 h at 37°C in cys⁻/met⁻ RPMI. The cells were then trypsinized, washed twice and adjusted to $4 \times 10^6$/ml in warm cys⁻/met⁻ RPMI. For continuous biosynthetic labeling, [$^{35}$S]-met *trans*-label (Amersham) was added (at 0.25 μCi/ml) and the cells were cultured for 2 h at 37°C at 7% $CO_2$. For timed experiments, cells were pulsed with [$^{35}$S]-met *trans*-label for 2 h then chased with RPMI containing an excess (2 mM) of cold met for appropriate time intervals. The labeled cells were then centrifuged and washed twice in RPMI and then resuspended at $5 \times 10^7$/ml in lysis buffer (consisting of 100 mM Tris–Cl pH 8.0, 140 mM NaCl, 0.25% $NaN_3$, 1% Triton X-100, 1% bovine hemoglobin, 5 mM iodo-acetamide, 1% aprotinin, 4 mM phenylmethylsufonyl fluoride (PMSF), 5 mM phenanthroline, 15 μM pepstatin, 560 μM N-tosyl-L-Phenylalanine chloromethyl ketone (TPCK), 5 mM benzamidine, 6.6 mM N-ethyl maleimide (NEM), 10 μM leupeptin, 270 μM N-tosyl-L-lysine chloromethyl ketone (TLCK), and 10 mM EDTA in TSA) and incubated for 60 min at 4°C. To facilitate cytolysis, the cell mixture was refluxed through a 27 g needle at the end of the incubation. The cell lysates were then centrifuged at 12,000*g* for 10 min (at 4°C) in a microfuge. The supernatant fluid was then precleared by a 2 h incubation at 4°C with quenched protein-A sepharose beads. The sepharose beads were separated and removed from the precleared lysates by centrifugation. Immuno-precipitation of solubilized MHC antigens was performed using specific antibodies (listed in Table IV) coupled to protein-A sepharose beads. Briefly, 40 μl of a 50% slurry of Ab-sepharose in dilution buffer (1% Triton X-100 and 1% Bovine hemoglobin in TSA, pH 8.0) was added to 100 μl of precleared lysate solution and 310 μl of dilution buffer. The reaction mixture was gently mixed on a rotator for 1.5 h at 4°C. Afterwards, the mixture was washed twice in dilution buffer, once in 50 mM Tris (pH 8.0) and once in 50 mM Tris–Cl (pH 6.8). The protein A-sepharose bound immunoprecipitates were then pelleted, dissociated by boiling for 4 min in 40 μl of 2X SDS sample buffer, fractionated by SDS-PAGE, then visualized and quantitated with a BioRad GS525 molecular imager system using an [$^{35}$S]-phosphoimager enhancement screen.

### Peptide Binding Assays

Binding of biotinylated P2 peptides to HLA DR β1*0301 expressing W-1s or LW-EBV-PBLs was determined in

TABLE IV Reagents used for immunoprecipitation of MHC Class II gene products involved in antigen processing and presentation

| Antibody specificity (human) | Clone | Host species/type | Source | Amount used per $5 \times 10^6$ cell equivalents |
|---|---|---|---|---|
| MHC Class I | W6/32 | Mouse/Mab | ATCC | 30 μl neat |
| MHC Class II | CR3/43 | Mouse/Mab | DAKO | 15 μl neat |
| Invariant chain | Pin-1 | Mouse/Mab | Dr. P. Cresswell | 15 μl neat |
| LMP-2/LMP-7 (*RING*12/*RING*10) | | Rb/Polyclonal | Dr. Patel/McDevitt | 30 μl neat |
| TAP-1/TAP-2 (*RING*4/*RING*11) | | Rb/Polyclonal | Dr. P. Cresswell | 5 μg/ml |
| DMB (*RING*7) | | Rb/Polyclonal | Dr. P. Cresswell | 10 μl 1:4 |

the following manner. A total of $5 \times 10^6$ EBV-PBLs or $10^7$ W-1s were suspended in a volume of 0.5 ml of RPMI containing 20% FBS, 2 mM L-glutamine, 50 μg/ml gentamicin, and 10 μM P2-biotin then incubated for 8 h at 37 EC in humidified 7% $CO_2$ atmosphere. After three washes in 10 ml of serum-free RPMI, the cell preparations were lysed, and immunoprecipitation with anti-HLA-DR bound to protein A sepharose beads was performed as described above. The immunoprecipitates were then washed twice in dilution buffer, once in 50 mM Tris-buffered saline, and then once in Tris–Cl pH 6.8. Next, a volume of 500 μl of streptavidin europium (Wallac, Turku, Finnland) diluted 1:500 in assay buffer was added to the washed samples which were then incubated on a rocker for 1 h at 4°C. The samples were then washed 7X in 50 mM Tris buffered saline. The washed pellets were then suspended in a volume of 100 μl of enhancement buffer and incubated on a rocker for 15 min at room temperature. The samples were then transferred to a 96 well cross-talk free microtiter plate (PE) and time-resolved Eu-phosphorescence was measured using a PE LS50B luminescence spectrophotometer (Perkin Elmer, Norwalk, CT).

### RESULTS

### Inability of MHC Class II (HLA-DRβ1*0301) Expressing W-1 Cell Line to Process and/or Present TT Antigen and the TT (830-843) Immunodominant HLA-DRβ1*0301-binding Peptide, P2

To evaluate the ability of W-1 cells to process exogenous antigen for presentation by MHC Class II, IFN-γ pretreated W-1s or autologous LW-EBV-PBLs were incubated with TT antigen as described, then used in proliferation assays with previously TT primed LW responder cells. While TT-primed unfractionated LW-PBMCs or purified T cells showed marked proliferative responses to TT antigen processed and presented by LW-EBV-PBLs, they showed no significant proliferative response to (identical) MHC class II expressing W-1s similarly pulsed with TT antigen (Fig. 1). The specificity

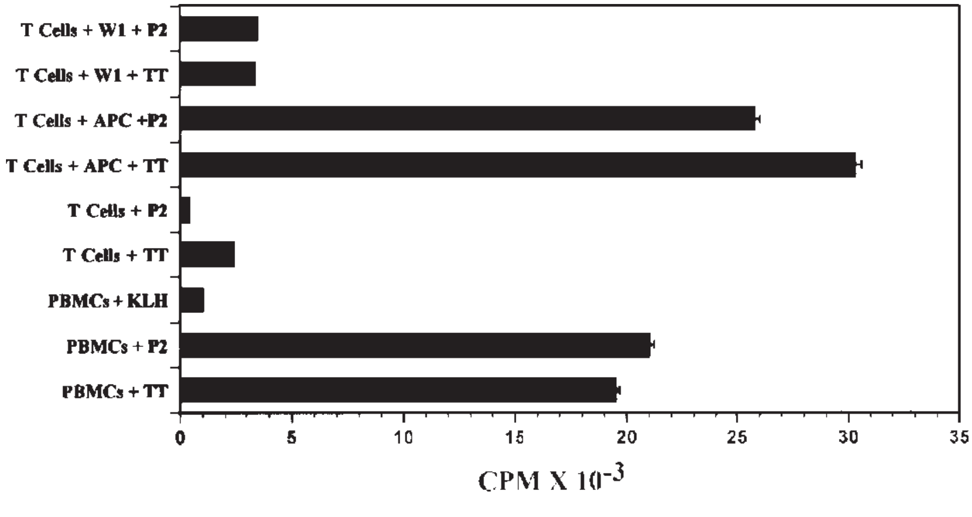

FIGURE 1 MHC class II restricted responses to TT antigen or P2 antigenic peptides presented by HLA DRβ1*0301 APCs. The abilities of IFN-γ pretreated W-1s and LW-EBV-PBLs to process and present TT antigen or to present synthetic P2 antigenic peptide to TT-primed unfractionated LW-PBMCs or purified LW-T cells were compared in 5 day proliferation assays as described in "Material and Methods" Section. Incorporation of [$^3$H]-thymidine was measured as the average CPM in triplicate wells. These results are representative of three separate experiments.

of the proliferative response was demonstrated by the absence of proliferation of LW-PBMCs in coculture with the control antigen KLH.

The ability of IFN-γ pretreated W-1s to present the synthetic HLA DR3-binding TT immunodominant peptide, P2, was also examined in these same sets of experiments. The APCs (IFN-γ pretreated W-1s or LW-EBV-PBLs) were pulsed with the immunodominant HLA-DRβ1*0301-binding TT 830-843 peptide (P2) as described. Again, we found marked proliferative responses by both the unfractionated PBMCs and the highly enriched T cells of LW to P2 peptide presented by autologous APCs while no significant proliferation was observed in aliquots of the same responder cells to the P2 peptide presented by MHC Class II expressing P2-pulsed W-1's (Fig. 1). The failure of W-1 cells to induce antigen specific T cell proliferation was not secondary to dose of antigen (TT or P2) or response kinetics (data not shown). Furthermore, APC's were clearly required since purified T cells did not proliferate when cultured alone with TT or the P2 peptide without APC's. Finally, non IFN-γ pretreated W-1 cells when pulsed with similar doses of TT or the P2 peptide also failed to induce significant T cell proliferation (data not shown).

### Ability of W-1 Cells to Process and Present Influenza a Matrix Peptide (M58-66) to HLA-A2 Restricted T Cells

MHC class I presentation of the defined influenza A matrix 1 protein was evaluated in a standard CTL assay as described in the "Materials and Methods" section. Vaccinia constructs containing the influenza A matrix 1 protein (M1-VAC) or the influenza A nucleoprotein (NP-VAC) were used to infect target cells for antigen loading into the class I processing pathway. Since W-1 cells constitutively express low but detectable levels of MHC Class I, we first examined the ability of W-1s without pretreatment with IFN-γ, to serve as targets for the PG CTL line specific for the M1 58-66 peptide presented by HLA-A*0201. As shown in Fig. 2A, W-1s infected with M1-VAC or a vaccinia construct containing the influenza A nucleoprotein (NP-VAC) as a negative control, were unable to be lysed by the PG CTL line. On the other hand, W-1s pulsed with the synthetic M1 58-66 antigenic peptide showed an average specific lysis between 40 and 50%, which was comparable to the results obtained when the autologous PG-EBV-PBL targets were used (Fig. 2C). When the W-1 targets were pretreated with IFN-γ (prior to infection with MI-VAC), an average specific lysis ranging from 20 to 30% was observed in the M1-VAC infected targets (Fig. 2B). The CTL response was M1 peptide specific since no lysis was observed in the control NP-VAC infected IFN-γ pretreated W-1 target cells. These values reflect a net specific lysis of approximately 50% of those obtained with PG-EBV-PBL targets infected with MI-VAC (Fig. 2C). The inability of W-1s to process and present M1 peptides for class I was shown not to be due to

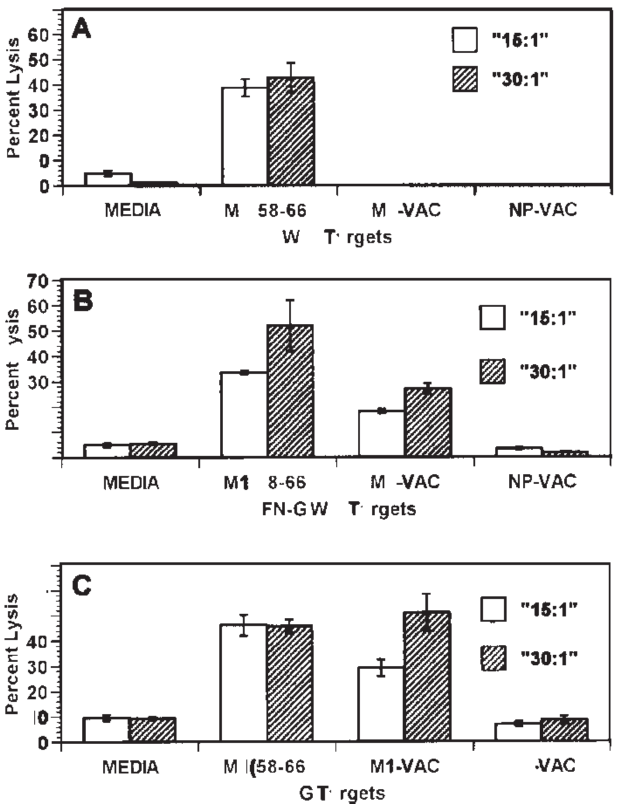

FIGURE 2 MHC class I restricted responses to influenza A matrix 1 protein the synthetic M1 (58-66) antigenic peptide presented HLA-A*0201 target cells. The abilities of W-1s (A), IFN-γ pretreated W-1s (B), and PG-EBV-PBLs (C) to process and present influenza A matrix 1 protein or to present M1 (58-66) antigenic peptide and serve as targets for the M1 peptide-specific cloned PG CTL cell line were compared in standard 5 h CTL assays as described in "Material and Methods" section. These results are representative of three separate experiments. To introduce antigen into the cytosol for processing by the class I pathway, target cells were infected with vaccinia constructs containing the influenza A matrix 1 protein (M1-VAC) or the influenza A nucleoprotein (NP-VAC). For other experiments target cells were incubated with the synthetic influenza A M1 (58-66) peptide before assaying for specific lysis by PG CTLs.

the failure of vaccinia to infect target cells since the presence of β-galactosidase in the cytosol of W-1 targets infected with control vaccinia constructs loaded with this indicator was confirmed colorimetrically by the degradation of 5-bromo-4chloro-3indoyl β-D-galactoside (X-gal) in lysed $^{51}$Cr-labeled W-1 targets cells (data not shown). Although, this is not a quantitative measurement of the level of expression of the influenza A matrix 1 protein or nucleoprotein within the cytosols of the vaccinia infected cells, the amount of substrate metabolized was essentially equivalent in all three target cell groups.

### Expression of Genes Encoding Components of the MHC Class I/II Antigen Processing Pathways

Within the MHC Class II locus, located on the short arm of human chromosome 6 are the genes which encode the MHC Class I heavy chain, the Class II α and β chains, and critical cellular components for the Class I and the Class II antigen processing pathways. Two subunits of the 20s proteasome, encoded by LMP-2 and LMP-7, and the transporters of antigenic peptides, encoded by TAP-1 and TAP-2, together process and transport endogenous antigens into the ER where they associate with nascent MHC class I molecules, which are subsequently transported to and expressed on the cell surface (Lehner and Cresswell, 1996; York and Rock, 1996). Though the role of DMA and DMB continues to be elucidated, DM has recently been shown to be involved in the exchange of processed antigenic peptides for processed invariant chain peptide (CLIP) on nascent MHC Class II molecules within the compartment for peptide loading (CPL) (Denzin and Cresswell, 1995; Sloan *et al.*, 1995; Weber *et al.*, 1996). Regulation of MHC Class II gene expression is coordinated by the Class II transactivator (CIITA) which is also located within the MHC Class II gene locus (Chang and Flavell, 1995). Although it is located on a separate chromosome, the gene encoding Ii is co-regulated with expression of the MHC Class II genes due to shared promoter/enhancer elements (Nordeng and Bakke, 1994). Deficient expression of any of these genes has been shown to result in defective or absent antigen presentation by MHC antigens (Mach *et al.*, 1996). Therefore, in order to assess antigen processing and presentation defects of the W-1 cell line from the perspective of gene expression, mRNA levels for each of these genes were determined by the semi-quantitative PCR method described above.

The constitutive expression of mRNA for genes involved in the class II antigen processing pathway were below detectable levels in W-1s, a finding consistent with the fact that W-1s do not constitutively express MHC class II. When W-1s were pretreated with IFN-γ to induce maximum expression of MHC class II, the transcription levels within the panel of class II genes were up regulated to a range of values all less than those constitutively expressed by the same genes in EBV-PBL controls. Figure 3A depicts the relative levels of expression of mRNA (copy equivalents) for MHC class II, CIITA, Ii, DMA, and DMB in IFN-γ W-1s as a percentage of the corresponding mRNA levels found in EBV-PBL controls. While mRNA levels in IFN-γ W-1s relative to EBV-PBLs are approximately 70 and 90% for DMB and DMA, respectively, they are represented at levels between 50 and 60% for Ii and CIITA, and fall significantly between 10 and 20% for MHC class II. These data suggest that although IFN-γ can up regulate transcription of multiple genes critical to the class II processing pathway, the markedly weak level of expression of MHC class II mRNA most likely has the strongest influence (at the transcriptional level) in limiting the ability of W-1s to process and present antigenic peptides for MHC class II. This interpretation is supported by results of FMF

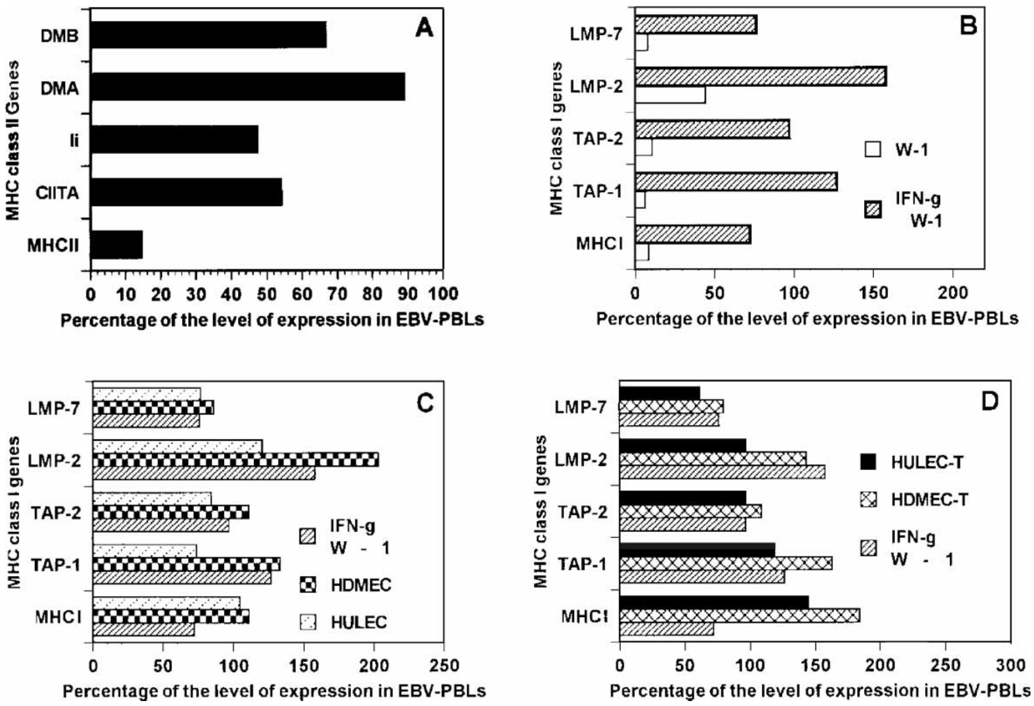

FIGURE 3 Comparison of mRNA levels of genes in the MHC class I/II antigen processing and presentation pathways in different APCs. Semi-quantitative measurements of mRNA level of genes involved in the class II pathway (MHC II, CIITA, Ii, DMA, and DMB) and the class I pathway (MHC-1, TAP-2, LMP-2, and LMP-7) were made by RT-PCR as described in "Materials and Methods" section. The levels of expression of mRNA (copy equivalents) from each cell line tested are expressed as a percentage of the constitutive expression of mRNA measured in the EBV-PBL controls. (A) Relative levels of class II pathway gene expression for IFN-γ W-1s. (B) Relative levels of class I pathway gene expression for W-1s and IFN-γ W-1s. (C) Relative levels of class I pathway gene expression for IFN-γ W-1s, primary human dermal microvascular endothelial cells (HDMEC), and primary human lung microvascular endothelial cells (HULEC). (D) Relative levels of class I pathway gene expression for IFN-γ W-1s, transformed HDMEC (HDMEC-T) and HULEC's (HULEC-T).

(data not shown) and pulse chase studies (detailed below) which confirm that the amount of MHC class II protein which is expressed on the cell surface or that can be immunoprecipitated from lysates of IFN-γ pretreated W-1s is only 10% of those values relative to EBV-PBLs.

To measure gene transcription within the class I antigen processing pathway, mRNA measurements of MHC class I, TAP-1, TAP-2, LMP-2 and LMP-7 genes were performed by RT-PCR as described. We have previously shown that the expression of MHC class I on W-1's cell surface is up regulated by IFN-γ (Ansari *et al.*, 1994). This finding is supported by measurements comparing the levels of the class I specific mRNAs found in W-1s and IFN-γ pretreated W-1s (Fig. 3B). An interesting observation was that IFN-γ pretreatment resulted in gene expression within W-1s that was approximately equivalent to (MHC I, TAP-2, LMP-7) or exceeding (TAP-1, LMP-2) that of EBV-PBLs. Since defects in antigen processing and presentation for MHC class I have been reported for several malignant and transformed cell lines (Seliger *et al.*, 1996), we chose (for a basis of comparison) to include measurements of constitutive levels of the specific mRNAs expressed in corresponding sets of both primary (Fig. 3C) and (SV40 large T antigen) transformed (Fig. 3D) microvascular endothelial cell lines. Compared to primary dermal or lung microvascular endothelial cells, the mRNA levels in W-1s were roughly equivalent with the exception of MHC class I which was slightly (but not significantly) lower. When IFN-γ pretreated W-1s were compared with (SV40) transformed dermal or lung microvascular endothelial cells, the level of mRNA for MHC class I was even higher in the immortalized cells than in the primary ECs. Thus, at least for gene transcription, we found no evidence of inhibition due to SV40 transformation.

### Pulse Chase Analysis of Components of the MHC Class I/II Antigen Processing Pathways

Having measured the relative levels of transcription of the genes involved in antigen processing and presentation, we next compared the relative levels and half lives of the translation products of the same set of genes from IFN-γ pretreated W-1s with the corresponding gene translation products from professional APCs. The antibodies used for these studies (listed in Table IV) were chosen for their ability to recognize and immunoprecipitate all the major isoforms of the individual components examined in our investigation: MHC I/II, Ii, LMP-7, TAP-1, TAP-2, and DMB. Our results showed (Fig. 4A) that the levels of immunoprecipitated gene products were much lower in the IFN-γ pretreated W-1 cell line, ranging from

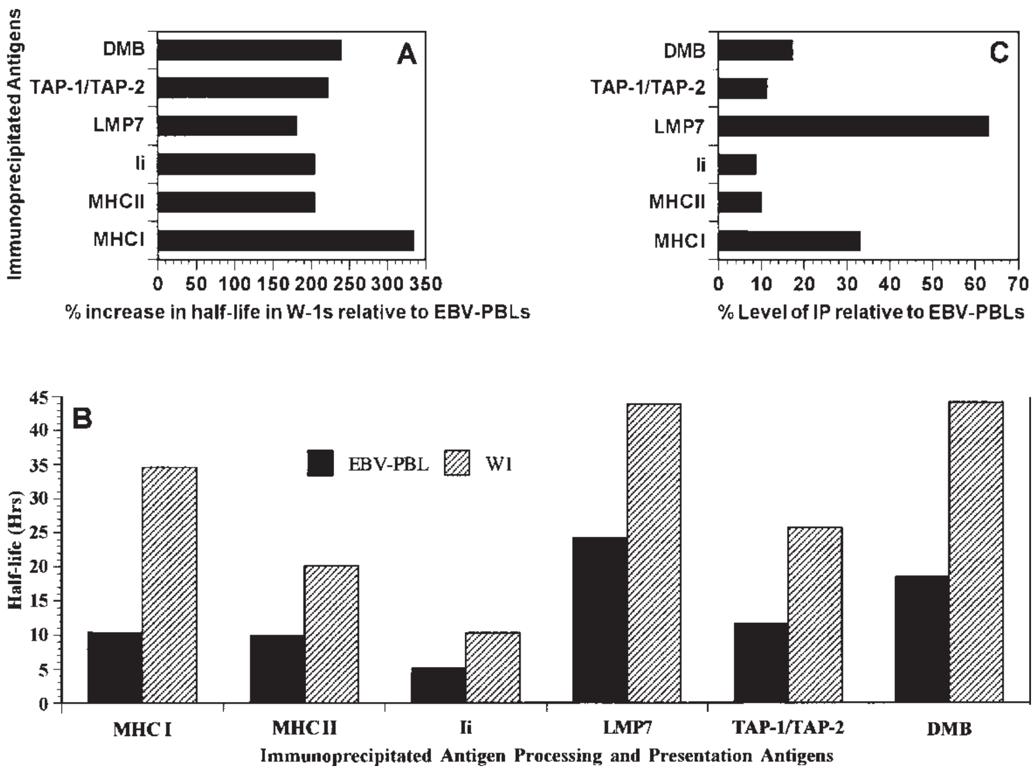

FIGURE 4 Relative levels and half-lives of immunoprecipitated proteins involved MHC class I/II antigen processing and presentation in IFN-γ W-1s and EBV-PBLs. (A) The relative levels of expression of the translation products of MHC class I/II, Ii, DMB, TAP-1/2, and LMP-7 genes for IFN-γ W-1s and EBV-PBLs were determined by immunoprecipitation with specific antibodies (listed in Table IV) as described in "Materials and Methods" section. The values for protein levels in IFN-γ W-1s are expressed as a percentage of the corresponding constitutive levels measured in EBV-PBLs. (B) The turnover rates (determined by pulse-chase analysis) for each of the immunoprecipitated gene translation products, expressed in half-lives (h), are compared for IFN-γ pretreated W-1s and EBV-PBLs. (C) The values for the half-lives of proteins immunoprecipitated in IFN-γ pretreated W-1s are expressed as a percentage of the corresponding constitutive values measured in EBV-PBLs.

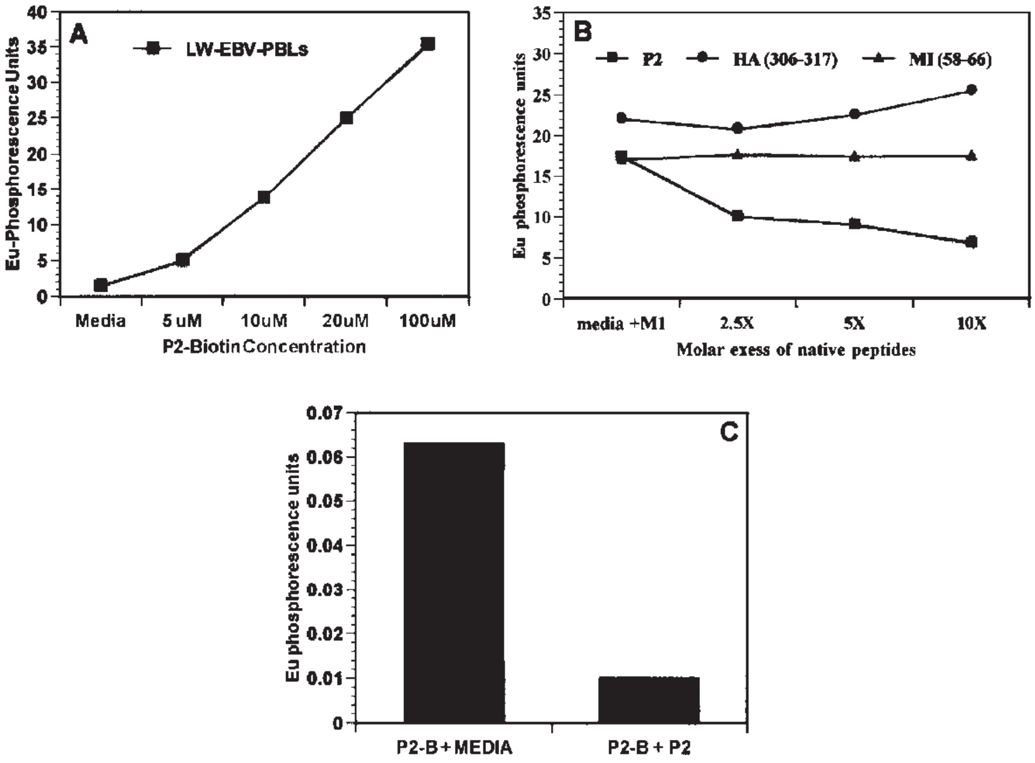

FIGURE 5 Binding of the synthetic TT (830-843) antigenic peptide (P2) to MHC class II on APCs. The binding of a biotinylated form of the P2 peptide (P2-B) to HLA DRβ1*0301 expressed on IFN-γ W-1s or LW-EBV-PBL controls was measured by the europium peptide binding assay described in "Materials and Methods" section. (A) Dose response of P2-B to LW-EBV-PBLs. (B) Competitive inhibition of P2-B binding to LW-EBV-PBLs by native P2 but not the HLA A*0201-binding peptide M1 (58-66) or the HLA DR1 binding peptide HA (306-317). (C) Levels and competitive inhibition of P2-B binding to IFN-γ W-1s by native P2 peptide.

approximately 10 (for MHC class II and Ii) to 60% (for LMP-7) of the corresponding levels observed in an equivalent number of JS-EBV-PBLs. Concomitantly, the half lives of all the same components measured were significantly increased in the IFN-γ pretreated W-1 cell line compared with the EBV-PBL controls (Fig. 4B,C). The values (based on the results of two separate experiments) of the percent increases in half-lives ranged from a low of approximately 180% for LMP-7 to a high of 330% for MHC Class I. Thus, the low levels of expression together with the slow turnover rates support the concept of a lethargic pace of antigen processing within the IFN-γ induced W-1 cell line and are consistent with the observed functional defects in antigen presentation by W-1's.

### Evaluation of P2 Peptide Binding to HLA-DR3

The W-1 cell line and the LW-EBV-PBL cell line, which were compared by their abilities to present P2 peptide to TT primed LW T cells, both share HLA DRβ1*0301 (as well as HLA DQβ1*0201) specificities (Table I). The potential of exogenously loaded P2 peptide to specifically bind to HLA DR in these two cell lines was confirmed by the Eu-based peptide binding assay described. A linear dose response to P2-peptide-MHC complexing was observed with HLA-DR immunoprecipitated from LW-EBV-PBLs pulsed with a biotinylated form of P2 peptide (Fig. 5A). In competitive inhibition assays unbiotinylated native P2 peptide at 5 molar excess was able to specifically inhibit binding of the biotinylated peptide bound to MHC class II expressed by LW-EBV-PBLs by approximately 50%, whereas the HLA-A*0201 binding M1 (58-66) peptide or the HLA DR1 binding HA (306-318) peptide were unable to inhibit P2-B peptide binding even when used at 10 molar excess concentrations (Fig. 5B). Similar results were obtained with parallel peptide-binding experiments performed with IFN-γ pretreated W-1 cells (Fig. 5C).

### DISCUSSION

In this study we have shown that the W-1 cell line is not able to effectively process and present the DR3 restricted TT(830-843) antigenic peptide, P2, and has only a moderate ability to process and present the HLA-A2 restricted influenza A M1 peptide. Our interest in pursuing this investigation was based on our previous findings that neither the W-1 cell line nor FHCM's induce primary or secondary alloresponses in T cells even after pretreatment with IFN-γ to induce their expression of MHC Class II (Ansari *et al.*, 1995a). Therefore, our focus in this study has been to explore the connections between the inability of W-1 cells to induce alloresponses and their intrinsic ability to process and present nominal antigens. It has been demonstrated that at least some T cell clones are capable of directly recognizing allo-MHC independent of bound peptide (Elliott and Eisen, 1990; Warrens, 1994). However, there is growing acceptance of the view that individual T cell clones recognizing multiple allogeneic determinants, formed by single processed oligomeric peptides complexed with MHC Class I/II molecules and displayed on the surface of the APC, determines the high precursor frequency of alloreactive T cells (Sherman and Chattopadhyay, 1993; Weber *et al.*, 1995). The requirement of peptide plus MHC for allorecognition implies that the diversity of peptides associated with MHC class I/II antigens governs the characteristic alloantigenic diversity of cells of different tissue lineages. Furthermore, Katz and Sant (1994) have proposed that the role of processed self-peptides may also explain why allorecognition can appear highly cell-type specific. Thus, restrictions in the ability to process and present peptides in association with MHC will limit the intrinsic allogenicity of an APC.

This view is supported by several lines of evidence. In an early report, Eckels *et al.* (1988) utilized a series of HLA-DR1 transfected human fibroblast lines to show that these DR-1 transfectants were able to present a synthetic influenza HA peptide to a panel of DR1 restricted T cell clones but were also unable to process or present the native intact virion. These same DR-1 transfectants were able to induce alloproliferative responses in only one out of a series of 25 specific alloreactive cloned T cell lines that recognized distinct allotypic determinants associated with HLA-DR1. An important implication from this study was that antigen processing is required for the creation of recognizable HLA-DR1 alloantigenic determinants on the APC. More recently, Cotner *et al.* (1991) have shown that HLA-DR3-specific T cell clones are unable to recognize and respond to HLA-DR3$^{+}$ B cell lines possessing mutations which prevent the formation of effective peptide MHC Class II complexes.

In context of these findings, we reasoned an alternative hypothesis for the observed lack of allogenicity of the W-1 cell line could be that cardiac myocytes possess a limited ability to process and present antigenic peptides for presentation by MHC resulting in the display of a number of allotopes insufficient to surpass a threshold leading to alloactivation or anergy that could be measured by conventional techniques. Such cell-type restrictions may be the result of the presentation of a unique set(s) of peptides processed from unique cell specific antigens, or uniquely processed from "house-keeping" antigens common to a broad variety of cell types, or both. It is our belief that in highly differentiated cells, e.g. cardiac myocytes, both of these types of restrictions may serve to limit the repertoire of allogeneic MHC:peptide conformers.

On a cellular level our study reveals that W-1s can present the influenza A M1(58-66) peptide to HLA-A*0201 restricted CD8+ T cell clones that are specific for the M1 peptide, but their moderate capacity to process and present the MI peptide is dependent on pre-treatment of the cell line with IFN-γ. This observation reveals three important points. First, it is clear that W-1's display a sufficient amount of MHC Class I (HLA-A2*0201) that is able to bind and present exogenously loaded M1 peptide for recognition by the HLA-A2-restricted PG CTL line. Second, W-1's also appear to display cell surface CSMs of sufficient quality and quantity to initiate antigen-specific CTL responses. However, it can be argued that the requirements for co-stimulation for productive allo-responses by naive T cells and cytolytic responses from a peptide-specific CTL line may differ. Third, pretreatment of W-1s with IFN-γ partially restores their ability to process the M1 peptide (Fig. 2C). The possibility that the (IFN-γ) induced expression of a novel cell surface molecule on W-1s is responsible for the observed lysis is unlikely since both untreated and IFN-γ pretreated W-1s pulsed with the MI peptide were able to be lysed equally well by PG CTLs (Fig. 2).

IFN-γ pretreatment did elevate gene expression for TAP-1/2 and LMP-2 to levels comparable to those seen constitutively in the EBV-PBL controls. However, mRNA levels measured for MHC-1 and LMP-7 were only moderately elevated. The modest increase in the expression of these two genes might present a partial explanation for the incomplete restoration of the ability to process the influenza A M1 peptide for HLA-A2-associated presentation. The precise role of LMP-2 and LMP-7 in the processing of antigenic peptides remains controversial. In 1992 reports of research from two separate laboratories were published describing reconstitution experiments in two different human B lymphoblastoid cell lines, T2 (Momburg *et al.*, 1992) and 721.174 (Arnold *et al.*, 1992), both having homozygous deletions in the MHC Class II region encompassing the genes encoding TAP-1/2 and LMP-2/7. Each group demonstrated transfection with genes encoding TAP-1/2 was both necessary and sufficient for surface expression of stable peptide MHC class I complexes by these cell lines. Furthermore, Momburg *et al.* (1992) demonstrated that reconstitution with TAP-1/2 alone was sufficient to restore the ability of the T2 line to process and present the influenza M1(58-66) peptide to an HLA-A2.1 restricted, M1 peptide-specific CTL clone, Q66.9. However, Fehling *et al.* (1994) have presented contradicting evidence by describing a knock-out strain of mice lacking LMP-7 which showed a markedly reduced ability to process and present the endogenous HY antigen. Furthermore, more recently, Cerundolo *et al.* (1995) were able to selectively restore the ability to process the M1(58-66) peptide for presentation to their M1-peptide specific CTL clone, JM 42, by introducing TAP-1/2 and LMP-7 into the antigen processing defective B cell line 721.174. These conflicting data might be reconciled by a role in which LMP-7 (and LMP-2) is required for the creation of new sets of antigenic peptides from viral (or self) antigens for presentation by MHC class I. This might also explain the partial restoration of W-1s to present the influenza A M-1 peptide. However, the under expression of

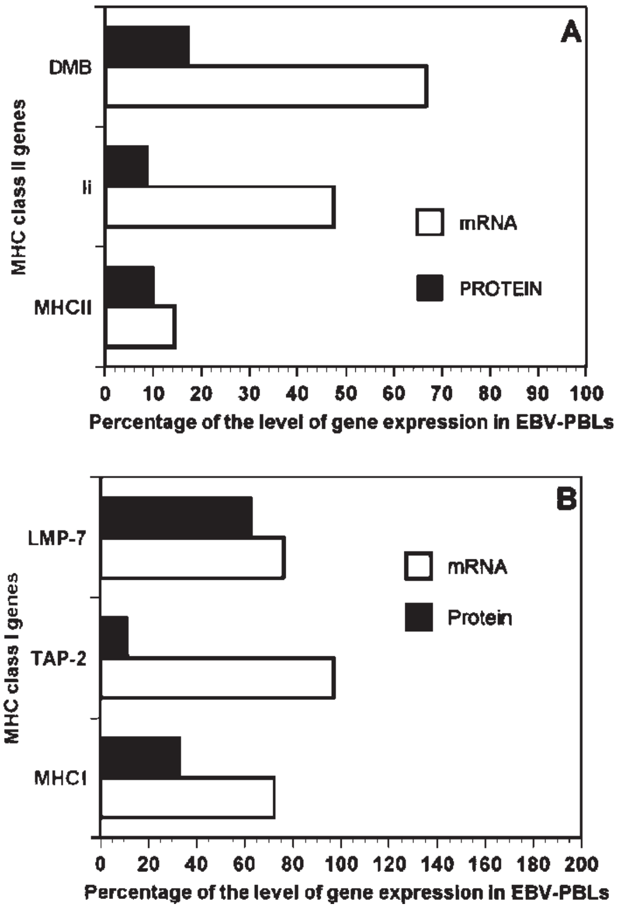

FIGURE 6 Comparison of transcription and translation of genes involved in the class 1 and class II antigen processing and presentation pathways. The levels of gene expression for IFN-γ W-1s are presented as a percentage of the corresponding levels in EBV-PBL controls. Gene transcription is expressed as levels of mRNA determined by semi-quantitative RT-PCR as described in "Materials and Methods" section. Gene translation is expressed as levels of protein antigens immunoprecipitated by the antibodies listed in Table IV as described. (A) Comparison of transcription and translation of the class II pathway genes MHC II, Ii and DMB. (B) Comparison of transcription and translation of the class II pathway genes MHC I, TAP-2, LMP-7.

other (IFN-γ) inducible components of the class I pathway (in W-1s) that were not a part of this study (e.g. chaperones, heat shock proteins, etc) may also be involved Williams and Watts (1995) as well as problems in gene translation.

Our investigation also revealed a significant difference in capacity between the class I and the class II antigen processing and presentation pathways in the W-1 cell line. Although W-1 cells respond to IFN-γ by expressing (SDS-stable) MHC class II (Ansari *et al.*, 1994), such pretreatment did not improve their ability to process or present the immunodominant TT 830-843 peptide to TT-specific HLA-DR3 restricted T cells (Fig. 1). To interpret these results in terms of the efficiency of gene expression within the MHC Class II locus, we compared the levels of transcription and translation relative to EBV-PBLs for selected genes involved in antigen processing and presentation for class I (Fig. 6B) and class II (Fig. 6A). When viewed this way, an interesting picture emerges. The inability of IFN-γ pretreated W-1s to present antigenic peptides in association with MHC class II, appears to be most directly correlated with its relatively weak expression of the genes encoding MHC class II and Ii. The levels of expression of Ii and MHC class II by IFN-γ W-1's were only 10–15% of those seen with EBV-PBL controls. This finding was confirmed at the level of gene transcription and translation (Fig. 6A) as well as for cell surface expression of MHC class II (by FMF-data not shown). However, the ability of HLA-DR3 expressing W-1's to specifically bind P2 peptide was measured to be only 0.5% of the amount able to bind to the HLA-DR3 expressing EBV-PBL controls (Fig. 5C). Therefore, a probable explanation for why IFN-γ W-1s are unable to present P2 to TT-primed T cells is their inability to efficiently bind the synthetic immunodominant TT (830-843) antigenic peptide. Furthermore, a relatively low density of naked MHC class II or the high affinity of self peptides associated with MHC Class II expressed on the cell surface, which could account for such inefficient binding, could be a result of inefficient antigen processing.

The results of our pulse-chase studies provide further evidence for inefficient antigen processing in IFN-γ W-1's. We found the half-lives for all of the gene products we examined within the MHC class II locus of W-1's to be at least 100% longer than the half-lives for corresponding gene products found in EBV-PBL controls (Fig. 4B). Furthermore, increasing the time for peptide or antigen pulsing with IFN-γ pretreated W-1's did not reconstitute their ability to induce antigen specific T cell activation (data not shown). While these results do not preclude the processing and presentation of antigenic peptides by IFN-γ W-1s, they do predict that the levels of processed antigenic peptides presented in association with MHC class I or class II would be proportionately lower than on professional APCs, e.g. EBV-PBLs. Both Ii and DM are involved in chaperoning nascent MHC Class II complexes through early and late endocytic compartments, where there may sample and acquire newly formed peptides (Castellino and Germain, 1995; Sherman *et al.*, 1995; Sanderson *et al.*, 1996). Restricted expression in these critical components of the class II pathway, as witnessed for W-1's (Fig. 6A), would likely influence the array of MHC II:peptide complexes (or allotopes) expressed on the cell surface. Likewise, the relative under representation of TAP-1/2 might limit the diversity of MHC 1: peptide allogeneic determinants in W-1's. It is also important to note that the under expression of other genes (not a part of this study) encoding proteases (e.g. cathepsin B and D), chaperones (e.g. calnexin), or other components involved in antigen processing for MHC class II may also contribute to antigen presentation defects observed in the W-1 cell line.

Another possible explanation for the lack of allogenicity observed in this cell line is insufficient second signaling due to under expression of critical CAM's/CSM's. Although FHCM's and W-1s do express important CSM's, e.g. LFA-3 and ICAM-1, we have shown that they do not constitutively express nor can they be induced to express critical CSM's, e.g. B7 (Ansari *et al.*, 1994). One of the trivial explanation for the disparities in the class I and II restricted responses of T cells to nominal antigens by W-1s could be that the expression of critical CSM's by W-1 are more stringently required for MHC class II restricted presentation than for class I. We have demonstrated that CHO cell transfectants expressing human HLA DR3 (but which do not express critical CSM's, e.g. LFA-3, ICAM-1, or B7) are able to present P2 peptide to HLA DR3$^+$ TT primed unfractionated PBMCs but not purified T cells (unpublished results). In contrast, IFN-γ pretreated W-1s were unable to present P2 peptide to either TT primed unfractionated PBMCs or purified T cells (Fig. 1), suggesting that requisite second signals for T cell responses to nominal antigen presented by W-1s can not be delivered in trans by third party autologous lymphocytes. Moreover, our unpublished data show that even after reconstitution with GPI-anchored B7, MHC class II expressing W-1 cells remain unable to present P2 peptide to TT primed responders. These findings lead us to conclude that factors such as the diversity and density of antigenic peptides as well as the affinity of MHC/TCR interactions, which collectively comprise first signals delivered in cognate interactions between T cells and APCs, may all be influenced by intrinsic limitations in antigen processing and presentation pathways in cardiac myocytes. The significance of second signals in the dialog between host immune cells and donor cardiac myocytes in models of chronic allograft rejection has been studied and are the subject of a separate publication.

### Acknowledgements

We thank Mr. Sukdev Brar for his help with PCR analysis. We would also like to thank Dr Peter Jensen and Dr Dominique Weber for peptides and research advice. Also, we would like to gratefully acknowledge the laboratory of Dr Peter Cresswell for antibodies and technical assistance in performing immunoprecipitation assays and semi-quantitative RT-PCR.

### References

Ades, E.W., Candal, F.J., Swerlick, R.A., George, V.G., Summers, S., Bosse, D.C. and Lawley, T.J. (1992) "HMEC-1: Establishment of an immortalized human microvascular endothelial cell line", *J. Investig. Dermatol.* **99**, 683.

Ansari, A.A., Sundstrom, J.B., Runnels, H., Jensen, P., Kanter, K., Mayne, A. and Herskowitz, A. (1994) "The absence of constitutive and induced expression of critical cell-adhesion molecules on human cardiac myocytes. Its role in transplant rejection", *Transplantation* **57**, 942.

Ansari, A.A., Sundstrom, J.B., Kanter, K., Mayne, A., Villinger, F., Gravanis, M.B. and Herskowitz, A. (1995a) "Cellular and molecular mechanisms of human cardiac myocyte injury after transplantation", *J. Heart Lung Transplant.* **14**, 102.

Ansari, A.A., Mayne, A., Sundstrom, J.B., Gravanis, M.B., Kanter, K., Sell, K.W., Villinger, F., Siu, C.O. and Herskowitz, A. (1995b) "Frequency of hypoxanthine guanine phosphoribosyltransferase (HPRT-) T cells in the peripheral blood of cardiac transplant recipients: a noninvasive technique for the diagnosis of allograft rejection", *Circulation* **92**, 862.

Arnold, D., Driscoll, J., Androlewicz, M., Hughes, E., Cresswell, P. and Spies, T. (1992) "Proteasome subunits encoded in the MHC are not generally required for the processing of peptides bound by MHC class I molecules", *Nature* **360**, 171.

Bednarek, M.A., Sauma, S.Y., Gammon, M.C., Porter, G., Tamhankar, S., Williamson, A.R. and Zweerink, H.J. (1991) "The minimum peptide epitope from the influenza virus matrix protein: extra and intracellular loading of HLA-A2", *J. Immunol.* **147**, 4047.

Bretscher, P. (1992) "The two-signal model of lymphocyte activation twenty-one years later", *Immunol. Today* **13**, 74.

Castellino, F. and Germain, R.N. (1995) "Extensive trafficking of MHC class II-invariant chain complexes in the endocytic pathway and appearance of peptide-loaded class II in multiple compartments", *Immunity* **2**, 73.

Cerundolo, V., Kelly, A., Elliott, T., Trowsdale, J. and Townsend, A. (1995) "Genes encoded in the major histocompatibility complex affecting the generation of peptides for TAP transport", *Eur. J. Immunol.* **25**, 554, [published erratum appears in Eur J Immunol May;25(5):1485].

Chang, C.H. and Flavell, R.A. (1995) "Class II transactivator regulates the expression of multiple genes involved in antigen presentation", *J. Exp. Med.* **18**, 765.

Cotner, T., Mellins, E., Johnson, A.H. and Pious, D. (1991) "Mutations affecting antigen processing impair class II-restricted allorecognition", *J. Immunol.* **146**, 414.

De Franco, A.L. and Coffman, R.L. (1995) "Lymphocyte activation and effector functions", *Curr. Opin. Immunol.* **7**, 303.

Denzin, L.K. and Cresswell, P. (1995) "HLA-DM induces clip dissociation from MHC class II alpha-beta dimers and facilitates peptide load", *Cell* **82**, 155.

Duquesnoy, R.J. and Demetris, A.J. (1995) "Immunopathology of cardiac transplant rejection", *Curr. Opin. Card.* **10**, 193.

Eckels, D.D., Sell, T.W., Long, E.O. and Sekaly, R.P. (1988) "Presentation of influenza hemagglutinin peptide in the presence of limited allostimulation by HLA-DR1 transfected human fibroblasts", *Hum. Immunol.* **21**, 173.

Elliott, T.J. and Eisen, H.N. (1990) "Cytotoxic T lymphocytes recognize a reconstituted class I histocompatibility antigen (HLA-A2) as an allogeneic target molecule", *PNAS* **87**, 5213.

Fehling, H.J., Swat, W., Laplace, C., Kuhn, R., Rajewsky, K., Muller, U. and Von, B.H. (1994) "MHC class I expression in mice lacking the proteasome subunit LMP-7", *Science* **265**, 1234.

Gotch, F., McMichael, A., Smith, G. and Moss, B. (1987) "Identification of viral molecules recognized by influenza-specific human cytotoxic T lymphocytes", *J. Exp. Med.*, 165.

Hayry, P., Alatalo, S., Myllarniemi, M., Raisanen-Sokolowski, A. and Lemstrom, K. (1995) "Cellular and molecular biology of chronic rejection", *Transplant. Proc.* **27**, 71.

Katz, J.F. and Sant, A.J. (1994) "T cell receptor recognition of MHC class II alloantigens is highly cell type dependent", *J. Immunol.* **152**, 1629.

Lawrence, E.C., Daneker, G.W., Waite-Rees, P.A., Lund, S.A., Candal, F.J., Bosse, D.C., Kuno, R., Kanter, K.R. and Ades, E.W. (1995) "Establishment of immortalized human lung microvascular endothelial cell lines", *Chest* **108**, 1265, (Abstract).

Lehner, P.J. and Cresswell, P. (1996) "Processing and delivery of peptides presented by MHC class I molecules", *Curr. Opin. Immunol.* **8**, 59.

Mach, B., Steimle, V., Martinez-Soria, E. and Reith, W. (1996) "Regulation of MHC class II genes: lessons from a disease", *Ann. Rev. Immunol.* **14**, 301.

Momburg, F., Ortiz-Navarrete, V., Neefjes, J., Goulmy, E., Van Y., De Wal, Spits, H., Powis, S.J., Butcher, G.W., Howard, J.C., Walden, P. and Hammerling, G.J. (1992) "Proteasome subunits encoded by the major histocompatibility complex are not essential for antigen presentation", *Nature* **360**, 174.

Morrison, J., Elvin, J., Latron, F., Gotch, F., Moots, R., Strominger, J.L. and McMichael, A. (1992) "Identification of the nonamer peptide from influenza A matrix protein and the role of pockets of HLA-A2 in its recognition by cytotoxic T lymphocytes", *Eur. J. Immunol.* **22**, 903.

Neefjes, J. (1995) "Antigen processing and presentation", *Res. Immunol.* **146**, 397.

Nordeng, T.W. and Bakke, O. (1994) "The bio-logical role of invariant chain (Ii) in MHC class II antigen presentation", *Immunol. Lett.* **43**, 47.

O'Sullivan, D., Arrhenius, T., Sidney, J., Del Guercio, M.F., Albertson, M., Wall, M., Oseroff, C., Southwood, S., Colon, S.M., Gaeta, F.C.A. and Sette, A. (1991) "On the interaction of promiscuous antigenic peptides with different DR alleles: identification of common structural motifs", *J. Immunol.* **147**, 2263.

Panina-Bordignon, P., Tan, A., Termijtelen, A., Demotz, S., Corradin, G. and Lanzavecchia, A. (1989) "Universally immunogenic T cell epitopes: promiscuous binding to human MHC class II and promiscuous recognition by T cells", *Eur. J. Immunol.* **19**, 2237.

Reece, J.C., Geysen, H.M. and Rodda, S.J. (1993) "Mapping the major human T helper epitopes of tetanus toxin: The emerging picture", *J. Immunol.* **151**, 6175.

Sanderson, F., Thomas, C., Neefjes, J. and Trowsdale, J. (1996) "Association between HLA-DM and HLA-DR *in vivo*", *Immunity* **4**, 87.

Seliger, B., Hohne, A., Knuth, A., Bernhard, H., Ehring, B., Tampe, R. and Huber, C. (1996) "Reduced membrane major histocompatibility complex class I density and stability in a subset of human renal cell carcinomas with low TAP and LMP expression", *Clin. Cancer Res.* **2**, 1427.

Shafer, W.M., Shepherd, M.E., Boltin, B., Wells, L. and Pohl, J. (1993) "Synthetic peptides of human lysosomal cathepsin G with potent antipseudomonal activity", *Infect. Immun.* **61**, 1900.

Sherman, L.A. and Chattopadhyay, S. (1993) "The molecular basis of allorecognition", *Ann. Rev. Immunol.* **11**, 385.

Sherman, M.A., Weber, D.A. and Jensen, P.E. (1995) "DM enhances peptide binding to class II MHC by release of invariant chain-derived peptide", *Immunity* **3**, 197.

Sloan, V.S., Cameron, P., Porter, G., Gammon, M., Amaya, M., Mellins, E. and Zaller, D.M. (1995) "Mediation by HLA-DM of dissociation of peptides from HLA-DR", *Nature* **375**, 802.

Sundstrom, J.B. and Ansari, A.A. (1995) "Comparative study of the role of professional versus semiprofessional or nonprofessional antigen presenting cells in the rejection of vascularized organ allografts", *Transplant. Immunol.* **3**, 273, [Review].

Villinger, F., Brar, S.S., Mayne, A., Chikkala, N. and Ansari, A.A. (1995) "Comparative sequence analysis of cytokine genes from human and nonhuman primates", *J. Immunol.* **155**, 3946.

Waite-Rees, P.A., Candal, F.J., Bosse, D.C., Ades, E.W., Swerlick, R.A., Kanter, K.R. and Lawrence, E.C. (1996) "Isolation, characterization, and immortalization of human pulmonary microvascular endothelial cells", *Am. J. Respir. Crit. Care Med.* **A570**, (Abstract).

Wang, Y.C., Neckelmann, N., Mayne, A., Herskowitz, A., Srinivasan, A., Sell, K.W. and Ahmed-Ansari, A. (1991) "Establishment of a human fetal cardiac myocyte cell line", *In Vitro Cell. Dev. Biol.* **27**, 63.

Warrens, A.N., Lombardi, G. and Lechler, R.I. (1994) "MHC and alloreactivity. Presentation and recognition of major and minor histocompatibility antigens", *Transplant. Immunol.* **2**, 103.

Weber, D.A., Terrell, N.K., Zhang, Y., Strindberg, G., Martin, J., Rudensky, A. and Braunstein, N.S. (1995) "Requirement for peptide in alloreactive CD4+ T cell recognition of class II MHC molecules", *J. Immunol.* **154**, 5153.

Weber, D.A., Evavold, B.D. and Jensen, P.E. (1996) "Enhanced dissociation of HLA-DR-bound peptides in the presence of HLA-DM", *Science* **274**, 618.

Williams, D.B. and Watts, T.H. (1995) "Molecular chaperones in antigen presentation", *Curr. Opin. Immunol.* **7**, 77.

York, I.A. and Rock, K.L. (1996) "Antigen processing and presentation by the class I major histocompatibility complex", *Ann. Rev. Immunol.* **14**, 369.

